# What barriers could impede access to mental health services for children and adolescents in Africa? A scoping review

**DOI:** 10.1186/s12913-023-09294-x

**Published:** 2023-04-06

**Authors:** Sabine Saade, Annick Parent-Lamarche, Tatiana Khalaf, Sara Makke, Alexander Legg

**Affiliations:** 1 Department of Psychology, American University of Beirut, P.O.Box 11-0236, Riad El-Solh/Beirut, 1107 2020 Lebanon; 2 Département de gestion des ressources humaines, Université du Québec à Trois-Rivières, 3351, boulevard des Forges, Trois-Rivières, QC G8Z 4M3 Canada; 3 Department of Psychology, CUNY John Jay College of Criminal Justice, 524 W 59th Street, New York, NY 10019 USA

**Keywords:** Africa, Mental health, Barriers, Children, Adolescents, Stigma, Knowledge

## Abstract

**Background:**

Few studies have examined the mental health needs of African children and teenagers. Based on this gap, this scoping review aims to identify barriers to mental health services, treatments and services sought, and where mental health services are received.

**Methods:**

To pursue the stated objectives, we searched the following databases a) PsycINFO, b) CINAHL, c) Medline, and d) Web of Science. The search yielded 15,956 records in total.

**Results:**

Studies included in this review were conducted in six African countries: Ethiopia, Mali, Egypt, South Africa, Nigeria, and Tunisia. The majority of the studies were conducted in South Africa (33.32%), followed by Ethiopia (25%), and Egypt (16.67%). In terms of treatments and services sought, both professional and traditional/alternative treatments were reported. The most frequently noted services were psychiatric treatments (25%), screening and diagnostic assessment (16.67%), as well as psychiatric and psychological consultations (16.67%). The most frequently reported treatment centers were psychiatric hospitals. As for treatment barriers, the three most frequently encountered barriers were: a preference for traditional/alternative and complementary treatments (33.33%), followed by stigma (25%), and a lack of knowledge/unfamiliarity with the mental health condition (25%).

**Conclusion:**

The results of this study are alarming due to the significant barriers to accessing mental health services coupled with the use of potentially harmful interventions to treat those mental health conditions. We hope this scoping review will help shed light on this important issue and help tomorrow’s generation reach its full potential.

## Background

According to the World Health Organization (WHO), mental health is defined as a state of well-being in which every individual realizes their own potential, can cope with the normal stressors of life, can work productively and fruitfully, and is able to contribute to their community [1]. Childhood and adolescence are critical periods in one’s lifespan. During these developmental periods, an individual is faced with many challenges they must overcome to successfully transition into the next period of their life [2]. In addition to representing critical periods of growth, these developmental periods could involve the inception and/or worsening of mental health conditions. Child and adolescent mental health conditions pose a public health challenge, impacting up to 20% of children worldwide [3]. The same finding extends to low and middle-income countries where studies on child mental health problems are scarce but are believed to be at least as prevalent as in high-income countries [4]. According to the WHO (2003), unmet mental health needs result in the loss of several billion dollars on health expenditures (including medical and pharmaceutical costs) and disability grants [3].

Early childhood experiences and mental health struggles could have serious and long-lasting consequences. Among those consequences are poor academic performance, troubled parent/child relationship, aggressive behaviors such as anger outbursts [5], lower academic achievement, violence, and poor reproductive and sexual health [6]. If left untreated, mental health difficulties could worsen, potentially resulting in more serious impairments (e.g., alcohol and drug use, depressed affect, and suicide attempts in adulthood) [7]. Beyond individual repercussions, childhood and adolescent mental health problems could lead to economic burden on the societal level [8]. Given the high prevalence rates of mental health conditions in childhood and adolescence [5], and the potentially negative consequences of such conditions, the need to access mental health services is heightened.

Despite the burden of mental illness, a number of children and adolescents suffering from mental health conditions are not accessing the help they need [6]. According to Whitney and Peterson [9], about half of the 7.7 million US children presenting with a mental health condition such as Attention-Deficit Hyperactivity Disorder (ADHD), anxiety, and depression did not receive psychological help. As for low and middle-income countries, the treatment gap is estimated at 90%. This gap is mainly due to poor detection and shortages of trained professionals [10, 11].

### The case of Africa

Even though some efforts, such as the decentralization of mental health care [12] have been made in some countries in Africa, significant challenges remain. Children in Africa are often exposed to known risk factors for mental health difficulties. Among those risk factors are, the Human Immunodeficiency Virus (HIV), malaria, tuberculosis, violence, crime, and low socioeconomic status, among other risk factors [13, 14]. In addition to these risk factors, child and adolescent mental health challenges could be exacerbated by system-wide variables, such as a shortage of trained professionals [10, 11]. In Africa, there are an estimated 1.4 mental health workers per 100,000 people compared to 9.0 average workers per 100,000 globally. In addition to the scarcity of mental health professionals, the region does not have enough psychiatrists, psychiatric hospital beds, and sufficient mental health outpatient coverage [14]. Faced with these challenges, the number of Africans who receive treatment for mental health difficulties is extremely low [14]. While the global annual rate of visits to mental health outpatient facilities is 1,051/100,000 population, this rate is estimated at 14/100,000 in Africa [15]. Approximately 98.8% of those in need of mental health services in Africa do not receive such mental health services [16]. In parallel to system-wide challenges, the role parents play in getting their children and teenagers mental health services is worth mentioning.

### The role parents play in getting their children the help they need

Parents play a prominent role in the mental health care of their children. Parents help their children access care, ensure treatment compliance, and provide support [17, 18]. Caregivers are often the first to detect early signs of mental illness and distress in their children. Additionally, children usually depend on their parents for access to mental health care, as they usually lack the means to do so independently [17]. In other words, parents are generally the gatekeepers to the ability of their children to access mental health care.

### Perceived gaps in the scientific literature and this study objectives

Shedding light on African childrens’ mental health is important for a number of reasons. In a span of 15 years (2000–2015), Africa’s population is estimated to have grown by 49%. Despite this population growth, the number of years lost to disability due to mental health and substance use disorders increased by 52% [19]. Although mental health needs have been explored in several countries [20, 21], there is a paucity of comprehensive reviews pertaining to the mental health needs of children and teenagers in African countries. Most research pertaining to mental health in Africa has focused on South Africa [22–24]. Expanding the search to include all countries in Africa could help paint a more accurate picture of mental health needs in an understudied context. While most previous studies focused on an adult population, child and adolescent mental health needs are still relatively unknown. Childhood and teenage years are crucial developmental years that can have potential long-term mental health consequences for the individual. Therefore, understanding access and barriers to mental health care during this sensitive developmental period is of particular importance. Understanding the mental health needs for children in Africa could help overcome potential barriers to accessing mental health services, as well as increase our understanding of where children and adolescents with mental health needs typically access services. This bottom-up approach is particularly useful in such contexts to inform mental health awareness campaigns, guide communities, and localize intervention efforts. Furthermore, most previous studies of child and adolescent mental health conducted in Africa have favored a qualitative approach. Although informative, qualitative data does not allow one to compare results obtained across studies and precludes us from drawing generalizable conclusions. Lastly, the challenges experienced by caregivers are relatively well documented in high-income countries [25], but less is known about such caregivers in low and middle-income countries [26, 27]. Given the key role parents play in the mental health care of their children, gathering their perspectives is important. Based on the perceived gaps in the scientific literature, this study aims to understand barriers to mental health services, the types of treatments received, and the location where such mental health treatments are provided. Although our initial search pertained to all countries in Africa, we ended up retaining studies pertaining to six countries. Although we focus on both children and adolescents in this review, we will use the term children to refer to both for ease of reading.

## Methods

In conducting this scoping review, we followed the following steps [28]:

- Stage 1: identifying the research question
- Stage 2: identifying relevant studies
- Stage 3: study selection
- Stage 4: charting the data
- Stage 5: collating, summarizing and reporting the results.

After having identified the research question (stage 1): understanding barriers to mental health services, the types of treatments received, and the location where such mental health treatments are provided, we searched the available literature.

### Search procedure

A scoping review was conducted on March 8, 2021 to identify the search terms and the databases to review. The search terms were meant to identify **barriers** (barrier* OR challenge* OR obstacle* OR impediment* OR hindrance* OR obstruction* OR hurdle* OR delay* OR access* OR isabil* OR refer*) **for children and adolescents under 18 years of age** (child* OR infant* OR baby OR babies OR toddler* OR kid* OR youth OR young OR teen* OR adolescen* OR minor OR student*) **with a mental illness** (“mental illness*” OR “mental disorder*” OR “mental difficult*” OR “mental problem*” OR “mental disturbance*” OR “psychological illness*” OR “psychological disorder*” OR “psychological difficult*” OR “psychological problem*” OR “psychological disturbance*” OR “psychiatric illness*” OR “psychiatric disorder*” OR “psychiatric difficult*” OR “psychiatric problem*” OR “psychiatric disturbance*” OR “isabi*r disorder*” OR “isabi*r difficult*” OR “isabi*r problem*” OR “isabi*r disturbance*” OR healthcare OR “psychological service*” OR “psychiatric service*” OR ADHD OR anxiety OR depress* OR “developmental delay*” OR autism OR autist* OR “intellectual isability*” OR OCD OR “communicative disorder*” OR “learning disorder*” OR “learning difficult*” OR “developmental coordination disorder*” OR “eating disorder*” OR mood* OR trauma* OR “Attention deficit hyperactivity disorder” OR “Developmental delay*” OR “Intellectual isability*” OR “Obsessive Compulsive Disorder*” OR “Learning disorder*” OR “Developmental coordination disorder*” OR “Eating disorder*” OR “Mood disorder*” OR “Psychological trauma” OR “Adjustment disorder*” OR “Schizophrenia” OR Dyslexia OR Dyscalculia OR “Psychotic disorder*” OR “Sexual and gender disorder*” OR “sexual disorder*” OR “gender disorder*” OR “Substance use disorder*” OR Dysgraphia OR “substance abuse” OR “substance misuse” OR “Psychological distress*” OR Asperger OR rett OR “Childhood disintegrative disorder*” OR “Pervasive Developmental Disorder*”) **in African countries or on the African continent** (Africa* OR Algeria* OR Egypt* OR Libya* OR Morroc* OR Sudan* OR Tunisia* OR Sahara* OR Burundi OR Comoros OR Djibouti OR Djibuti OR Eritrea* OR Ethiopia* OR Kenya* OR Madagascar OR Malawi OR Mauritius OR Mozambique OR Rwanda* OR Seychelles OR Somal* OR Sudan* OR Tanzania* OR Uganda* OR Zambia* OR Zimbabwe* OR Angola* OR Cameroon OR Chad OR Congo* OR Gabon OR Principe OR Botswana* OR Eswatini OR Lesotho OR Namibia* OR Benin OR Gambia* OR Ghana* OR Guinea OR Bissau OR Liberia* OR Mali OR Mauritania* OR Niger* OR Nigeria* OR Senegal* OR Togo OR “British Indian Ocean Territor*” OR “French Southern Territor*” OR “Middle Africa*” OR “Sao Tome” OR “Burkina Faso” OR “Cabo Verde” OR “Cape Verde” OR “Cote d’Ivoire” OR “Saint Helena” OR “Sierra Leone*”). We limited our search to scientific articles published between January 1, 2011, until March 8, 2021.

We searched the following databases a) PsycINFO, b) CINAHL, c) Medline and d) Web of Science. The search yielded 1,455 records from PsycINFO, 953 records from CINAHL, 5,289 records from Medline, and 8,259 records from Web of Science. In total, 15,956 records were retrieved and downloaded onto Endnote before transferring them to an Excel file. After duplicates were removed, we were left with 11,980 unique records to screen.

### Inclusion criteria

To be included in the review, studies had to meet the following criteria:

- Peer-reviewed
- Published in English, French, or Arabic
- Published in the past 10 years
- Included a sample of participants aged 18 years old or younger
- Conducted in Africa
- Measured a psychological disorder or symptom

### Exclusion criteria

Studies were excluded if they were:

- Books and/or book chapters
- Dissertations
- Theses
- Case studies
- Systematic reviews/Literature reviews
- Meta-analyses
- Reports
- Qualitative studies
- Not published in the past 10 years
- Included participants over 18 years old
- Not conducted in Africa
- Pertained to medical rather than mental health conditions. Examples of such medical conditions included HIV or birth-related complications.
- Measured personality disorders, dissociative disorders such as dissociative identity disorder or dissociative fugue, or food addictions. We decided to exclude studies focusing on personality disorders due to the fact that such disorders cannot be diagnosed before the age of 18 [29].

### Data extraction

**Fig. 1:**
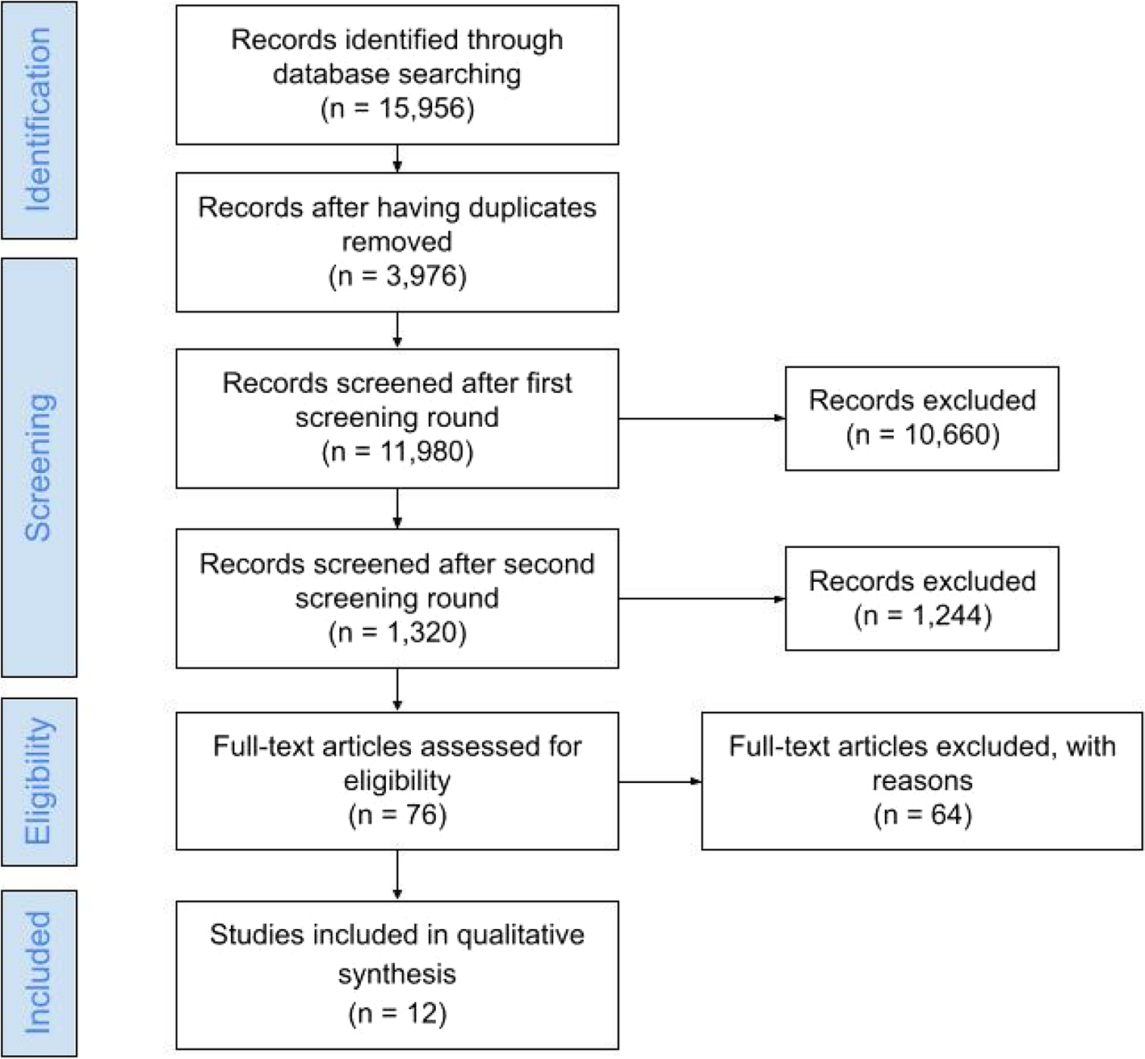
PRISMA flow diagram [30]

After all records had been retrieved, screening was conducted in a series of steps (stage 2: identifying relevant studies and stage 3: study selection; Fig. 1). Database searching yielded 15,956 records. After screening, 3,976 duplicate records were removed. The first step consisted of screening the titles and the abstracts of the remaining 11,980 records. Based on this first screen, 10,660 records were excluded for not meeting the eligibility criteria of the current study. The remaining 1,320 records were screened a second time (title and abstract), among which another 1,244 were excluded for not meeting the eligibility criteria. Next, each of the remaining 76 studies was read in full and was independently screened by two raters. In case of doubt about article eligibility, the first author made the final decision. Based on this screening process, 64 studies were excluded due to not meeting the eligibility criteria of the current study. In the end, 12 studies met the objective of the current study and were included in our review.

The large heterogeneity in sample sizes, participants’ age, methodology adopted, measurement tools, treatment and service names, and whether the researchers evaluated mental health symptoms or mental health diagnoses precluded us from conducting a meta-analysis. Figure 1 illustrates the results of the search and of the screening and selection process for the inclusion of studies in our review.

The following information was extracted from each study (stage 4, charting the data):

- Author(s) of the study
- Title of the study
- Year of publication
- Type of study
- Study Design
- Measurement tools
- Analyses used
- Barriers to mental health services
- Type of treatment/s received
- Treatment center/Location

Each study was evaluated for a risk of bias based on the Checklist for Assessing the Quality of Quantitative Studies [31] (Table 1). This evaluation tool was selected due to the comprehensiveness and inclusiveness of all criteria considered relevant in the evaluation of quantitative studies. According to the Checklist for Assessing the Quality of Quantitative Studies [31], a study was considered to have a low bias if:

- The question or the objective was sufficiently described.
- The design was evident and appropriate to answer the study question.
- The method of subject selection (and comparison group selection, if applicable) or source of information/input variables (e.g., for decision analysis) was described and appropriate.
- The subject (and comparison group, if applicable) characteristics or input variables/information (e.g., for decision analyses) were sufficiently described.
- The outcome and (if applicable) exposure measure(s) were well-defined and robust to measurement/misclassification bias. The means of assessment were reported.
- The sample size was appropriate.
- The analysis was described and appropriate.
- Some estimate of variance (e.g., confidence intervals, standard errors) was reported for the main results/outcomes (i.e., those directly addressing the study question/ objective upon which the conclusions are based).
- The authors controlled for confounding variables.
- The results were reported in sufficient detail.
- The results supported the conclusions.

**Table 1:** Methods and analyses used, barriers to mental health services, type of treatment/s received, treatment center and studies’ risk of bias score

**Author(s)**	**Title**	**Year**	**Methods and Analyses Used**	**Barriers to Mental Health Services**	**Type of Treatment/s Received**	**Treatment Center/ Location**	**Risk of Bias Score**
Abera et al. [32]	Parents’ Perception of Child and Adolescent Mental Health Problems and Their Choice of Treatment Option in Southwest Ethiopia	2015	*Type of Study:* Quantitative *Study Design:* Cross-sectional design *Measurement Tools:* Socio-demographic questionnaire and a checklist adapted from the Strengths and Difficulties Questionnaire (SDQ) Analyses Used: Descriptive statistics and inferential statistics (Chi-square and multivariate logistic regression analysis)	*Expectations About Care Services:* 69.2% of parents indicated a lack of availability of modern mental health care outside the big cities *Treatment Modalities:* 92.7% of parents indicated that religious or spiritual healers were available nearby for any psychiatric problems their children might present. “Holy water, Rukiya (Holy Quran-based religious treatment), praying at home and in the church by religious people were the most commonly mentioned religious and spiritual modalities of treatment for psychiatric disturbance in children *Education:* Compared to *better-educated* parents, those who were illiterate and *less educated* (less than 9 Y of schooling) were approximately five times *more likely* to prefer traditional treatment options (AOR = 4.48; CI = 2.82, 7.12) *Occupation:* Individuals with an occupation as a housewife or a farmer were 1.5 times *more likely* to prefer traditional treatment compared to merchants and individuals with other occupations (COR = 1.51; CI = 1.03, 2.29) *Religion:* Compared to Muslims, Coptic Christians were two times *more likely* to prefer traditional treatment (COR = 1.71; CI = 1.14, 2.55) *Beliefs About Causes:* Individuals who believed that mental illness had supernatural causes were 4.3 times *more likely* to prefer traditional treatment compared to their counterparts (AOR = 4.33; CI = 1.86, 10.09)	NR	NR	20
Abiodun et al. [33]	Detecting Child Psychiatric Disorders During Routine Clinic Work: A Pre-Interventional Study of Primary Care Physicians in Ilorin, Nigeria	2011	*Type of Study:* Quantitative *Study Design:* Cross-sectional design *Measurement Tools:* Parents’ version of the Child Behavior Questionnaire (CBCQ), Rutter Scale A2, and the Children’s version of the Schedule for Affective Disorders and Schizophrenia (SADS) *Analyses Used:* Descriptive analysis and inferential statistics (Chi-square analysis)	*Assessment and Detection Issues:* Primary care physicians were not able to correctly detect child psychiatric cases to refer them to treatment	NR	Child and adolescent psychiatric clinic, Pediatric Clinic of the Department of Family Medicine, University of Ilorin Teaching Hospital	20
Bourgou et al. [34]	Tunisian Mothers’ Beliefs About Their Child’s First Psychotic Episode	2012	*Type of Study:* Quantitative *Study Design:* Cross-sectional design *Measurement Tools:* Questionnaire developed by the research team *Analyses Used:* Descriptive analysis and inferential statistics (correlational analysis)	*Stigma:* 70% of mothers indicated that stigma related to psychiatry and hospitals, in general, constituted barriers to seeking mental health treatment *Fear Related to Medications:* Fear of secondary effects associated with psychiatric treatments (50%) and fear of dependence on psychotropic medication (31.8%) were identified as barriers to seeking mental health treatment *Patient Refusal:* Patient refusal to seek help was identified as a barrier to seeking mental health treatment (40.9%) *Lack of Access:* Lack of access to healthcare was identified as a barrier to seeking mental health treatment (36.4%) *Financial Difficulties:* Financial difficulties were identified as a barrier to seeking mental health treatment (31.8%) *Disease Denial:* Disease denial was identified as a barrier to seeking mental health treatment (4.5%)	Psychiatric treatment	Child psychiatry unit of Razi Hospital	17
Burnhams et al. [35]	Social Service Offices as a Point of Entry into Substance Abuse Treatment for Poor South Africans	2012	*Type of Study:* Quantitative *Study Design:* Cross-sectional design *Measurement Tools*: Questionnaire developed by the research team (some of the items were taken from the SACENDU data collection tool as well as the Treatment Services Audit questionnaire used to audit substance abuse treatment services in South Africa) *Analyses Used:* Descriptive analysis and inferential statistics (Chi-square and paired sample T-tests)	*Age:* Age was + *vely* associated with seeking specialized services. Clients who used substances and were seeking social welfare services were significantly *younger* than those attending specialist drug treatment facilities (*t* = 6.44 (*df* = 16,773), *p* < .001). The former was relatively young (*M* = 25.2 Y; *SD* = 11.98), and 42.4% of the sample was between 15 and 19 Y of age *Race:* Significantly *more Black African* (Chi-square = 5.12 (*df* = 1), *p* < .02) *and Colored clients* (Chi-square = 68.45 (*df* = 1), *p* < .001) sought services in social welfare offices compared to seeking specialist treatment *Area:* Close to two-thirds of persons using substances who sought social welfare services were from rural districts *Education:* A significantly *greater* proportion of clients from the social welfare services system did not complete high school compared to clients who attended specialized treatment (Chi-square = 414.20 (*df* = 1), *p* < .001) *Employment:* A significantly *smaller* number of clients who use substances attending social welfare services were employed compared to those who attended specialized treatment (Chi-square = 16.75 (*df* = 1), *p* < .001)	Social services Specialist substance abuse treatment	Specialist substance abuse treatment facilities	22
Erasmus et al. [22]	Onset of Intervention for Learners in Autism-Specific Government-Funded Schools in South Africa	2021	*Type of Study:* Quantitative *Study Design:* Cross-sectional design *Measurement Tools*: Questionnaire developed by the research team *Analyses Used:* Descriptive analysis and inferential statistics (non-parametric tests to explore associations and correlations between variables. Specific tests not indicated)	*Parental Concern:* There were significant, + *ve* associations between school admission age and age at which parents were concerned (*r* = .197; *p* = < .000), age at first assessment (*r* = .210; *p* = < .001), and age at diagnosis (*r* = .239; *p* = < .000). + *ve* associations were found between age at assessment and age at parental concern (*r* = .563; *p* < .000), age at diagnosis (*r* = .584; *p* < .000), and age at admission to the autism-specific school (*r* = .210; *p* < .001). Thus, the *later* caregivers became concerned (*M* = 25.2 months), the *later* the age at first assessment (*M* = 34.7 months), the age of ASD diagnosis (*M* = 46.6 months), and the age at admission to the autism-specific school (*M* = 6.8 Y) *Lack of Awareness/Unfamiliarity with ASD:* Caregivers were not aware of early symptoms of ASD, as shown by the *later* age at parental concern *Relationship to Patient:* Being the father, caregiver, or guardian was -*vely* associated with concern over the child’s development *Citizenship:* Being a South-African citizen was* -vely* associated with caregiver concern about their child’s development. South African caregivers became concerned about their child’s development *later* than caregivers from countries within and outside Africa *Education:* Level of parental education was *-vely* associated with being concerned about their child’s development *Type of Service Sought:* Instead of seeking help from professionals specializing in ASD diagnosis, caregivers looked for help first from allied health professionals providing intervention for DD	Educational intervention	Autism-specific government-funded schools	18
Girma and Tesfaye [36]	Patterns of Treatment Seeking Behavior for Mental Illnesses in Southwest Ethiopia: A Hospital Based Study	2011	*Type of Study:* Quantitative *Study Design:* Cross-sectional design *Measurement Tools:* Questionnaire adapted from the WHO encounter form for pathways to care and supplemented with items that assess the perception of patients on mental illness developed from the Good pathway model *Analyses Used:* Descriptive analysis and inferential statistics (logistic regression)	*History of Suicide Attempt:* Compared to people with no history of suicide attempts, people who had attempted suicide were *less likely* to seek treatment early [OR-.2, 95% CI (.09, .65)]	Psychiatric treatment	Jimma University Specialized Hospital	20
Gobrial et al. [37]	Mind the Gap: The Human Rights of Children with Intellectual Disabilities in Egypt	2012	*Type of Study:* Quantitative *Study Design:* Cross-sectional design *Measurement Tools:* Questionnaire developed from the United Nations Convention on the rights of persons with disabilities and the UK’s equality Act *Analyses Used:* Descriptive analysis	*Lack of Awareness of Human Rights of ID:* “Four out of five respondents (83%) reported that they had rarely or hardly ever heard or read about human rights in relation to children with disabilities; only a few (4%) of the respondents reported that this issue was ever discussed in the media. Nevertheless, a very high percentage (85%) expressed interest in knowing more about the human rights of children with IDs and three out of four (75%) believed that this group of children should have access to the same basic rights as other children” *Role of Government:* The Egyptian government’s role in providing access to human rights for children with ID was criticized. Almost two-thirds (62%) of the respondents mentioned that the government is not doing enough to bring attention to the rights of children with ID, while two-thirds (67%) indicated that the government did not protect children with ID from discrimination and its effects	NR	Local parent support groups Charities Day care centers for children with disabilities	18
Hussein et al. [38]	Pathways to Child Mental Health Services Among Patients in an Urban Clinical Setting in Egypt	2012	*Type of Study:* Quantitative *Study Design:* Cross-sectional design *Measurement Tools:* Questionnaire developed by the research team, Arabic version of Stanford-Binet Intelligence Scale, Gilliam Autism Rating Scale (GARS), Kiddie Schedule for Affective Disorders and Schizophrenia-present and lifetime Version (SADS), Fahmy and Sherbini’s social classification scale *Analyses Used:* Descriptive analysis	NR	Child psychiatry, pediatric psychiatric consultations	Government-operated, urban, outpatient mental health clinic of child psychiatry	20
Pillay and Siyothula [23]	Intellectual Disability Examinations and Social Context Variables Among Patients of Low Socioeconomic Status	2011	*Type of Study:* Quantitative *Study Design:* Cross-sectional design *Measurement Tools*: Questionnaire developed by the research team and colored progressive matrices *Analyses Used:* Descriptive analysis and inferential statistics (correlation and logistic regression)	*Transportation Costs:* Transportation costs to attend the consultation ranged between South African Rand (R) R0.00 and R240.00 (+ U.S. $35; *M* = R38.50; *SD* = U.S. $5). 26% of caregivers mentioned they faced financial difficulties to the extent that they had to go without food on some days	Psychological consultations ID screening assessments	Various state clinical psychology clinics served by the authors’ institution	22
Rajcumar and Paruk [39]	Knowledge and Misconceptions of Parents of Children with Attention-Deficit Hyperactivity Disorder at a Hospital in South Africa	2020	*Type of Study:* Quantitative *Measurement Tools:* Questionnaire developed by the research team and Knowledge of Attention Deficit Disorder Scale (KADDS) *Study Design:* Cross-sectional design *Analyses Used:* Descriptive analysis	*Parental Knowledge and Misconceptions of Their Child’s ADHD Diagnosis:* Most parents knew a bit about their child’s ADHD diagnosis despite holding multiple misconceptions about its treatment and related factors: “92.4% believed that reducing sugar or food additives were effective to reduce symptoms; 78.5% that treatments focusing on punishment reduced the symptoms; 67.1% that prolonged use of stimulant medications leads to increased addiction (i.e., drug, alcohol) in adulthood” *Stigma:* Most parents (*n* = 60, 75.9%) reported experiencing stigma related to their child’s mental health condition *Treatment Pathway:* 32.9% of parents consulted a traditional healer for their child, and 84.6% did so before seeing a medical doctor	NR	Public sector outpatient psychiatric hospital	20
Sangare et al. [40]	Health Facility-Based Prevalence and Potential Risk Factors of Autism Spectrum Disorders in Mali	2019	*Type of Study:* Quantitative *Study Design:* Cross-sectional design *Measurement Tools:* Questionnaire developed by the research team and review of the outpatient medical charts of 12,000 children aged 3–14 years old treated at the study sites from 2004 to 2014 *Analyses Used:* Descriptive analysis	NR	NR	All the public and private health facilities and organizations involved in the diagnosis and management of ASD	22
Tilahun et al. [25]	Stigma, Explanatory Models and Unmet Needs of Caregivers of Children with Developmental Disorders in a Low-Income African Country: A Cross-Sectional Facility-Based Survey	2016	*Type of Study:* Quantitative *Study Design:* Cross-sectional design *Measurement Tools:* Questionnaire developed by the research team with some of its items drawn from the adapted version of the Family Interview Schedule (FIS) *Analyses Used:* Descriptive analysis and inferential statistics (stepwise multiple linear regression and non-parametric Mann–Whitney tests)	*Support Sought:* More than half of the caregivers indicated they first looked at traditional places for help (*n* = 56; 54.9%), while less than half of the participants visited a biomedical institution first (*n* = 46; 45.1%) Other types of help sought prior to coming to the current child mental health clinic were a hospital (*n* = 83; 81.4%) and/or a private clinic (*n* = 27; 26.5%), traditional institutions, religious healing centers (e.g., holy water (*n* = 53; 52.0%), a church or priest (*n* = 35; 34.3%) or other traditional healers Caregivers used both “biomedical treatments (tablets (*n* = 40; 39.2%) or injections (*n* = 4; 3.9%) received through a health facility) and traditional interventions (prayer *n* = 48; 47.1%), itab (a written script tied on the arm or neck; *n* = 8; 7.8%), slaughtering a sheep (*n* = 4; 3.9%), or fumigating (making excessive use of smoke by burning incense;* n* = 2; 2.0%) as treatment interventions. A subgroup of caregivers also indicated they had used beating (*n* = 19; 18.6%) or chaining (*n* = 9; 8.8%) to manage their child” *Stigma:* Most caregivers experienced stigma: 43.1% were concerned about being treated differently, 45.1% felt shame related to their child’s condition, and 26.7% tried to keep their child’s condition secret. Stigma was *-vely* associated with looking for professional treatment. Stigma was significantly *higher* in caregivers who looked for traditional help (*p* < .01)	Specialist expertise in child DD Out-patient child mental services for children with ASD and/or ID, including diagnostic assessment, medication where appropriate, signposting to available community services, and ongoing follow-up	Child mental health clinics at medical college hospitals	20

Given that no intervention or treatment study was included in our review, we did not score the following three items:

- If random allocation to the treatment group was possible and described.
- If interventional and blinding of investigators to intervention was possible and reported.
- If interventional and blinding of subjects to intervention was possible and reported.

Each item was scored as follows: A score of 2 was allocated to a “Yes,” a score of 1 was allocated to a “Partial yes,” and a score of 0 was allocated to a “No”. In total, each study was rated using this 14-item questionnaire with the exception of the three items pertaining to intervention/treatment studies. Thus, each study’s risk of bias was evaluated based on 11 items. A low score indicated a high risk of bias, while a high score indicated a low risk of bias. The minimum bias score was 0 and the maximum was 22.

In the following paragraphs, we report percentages that were computed as follows:

(Number of studies meeting a relevant criterion/Number of studies included in this review)*100. For instance, when computing the percentage of studies reporting a preference for traditional/alternative and complementary treatments, we used the following formula: (4 studies/12 total studies)*100 = 33.32%.

## Results

In total, studies included in this review were conducted in six African countries: Ethiopia, Mali, Egypt, South Africa, Nigeria, and Tunisia. The majority of the studies were conducted in South Africa (33.32%), followed by Ethiopia (25%), and Egypt (16.67%). Studies included in this review had a bias risk score ranging between 17 and 22 (stage 5, collating, summarizing and reporting the results).

### Mental health conditions and mental health challenges

Participants in the included studies suffered from various mental health conditions. Among these conditions are: Autism Spectrum Disorders (ASD), Intellectual Disability (ID), Substance Use Disorders (SUD), Developmental Delays (DD), ADHD, Conduct Disorders (CD), Major Depressive Disorder (MDD), anxiety, schizophrenia, schizophreniform disorder, schizoaffective disorder, nocturnal enuresis, and Bipolar Disorder (BD). Other mental health concerns included emotional, peer-relationship, and social difficulties.

### Treatments/services sought

In terms of treatments and services sought, the most frequently reported services were psychiatric treatments (25%), screening and diagnostic assessments (16.67%), and psychiatric and psychological consultations (16.67%). Other services included referrals to available community services, follow-ups, specialist treatments such as those tailored to DD, social services, and educational interventions. Treatments and services sought were described differently across studies with varying levels of detail.

### Treatment centers/treatment locations

The three most frequently reported treatment centers were psychiatric hospitals (41.67%), mental health clinics (25%), and public and private health facilities (16.67%). Other treatment locations included government-funded schools (8.34%), local support groups, charities, and daycare centers (8.34%). The reason why psychiatric hospitals were the most frequented treatment center is likely attributed to the severity of the mental health conditions.

### Barriers to seeking mental health services

Reported barriers to mental health services are essential in understanding the unmet mental health needs of African children and adolescents. A better understanding of these mental health gaps will hopefully inform public health policy and provide the future generation with the help it desperately needs.

The most frequently reported barrier to accessing mental health services was a preference for traditional/alternative and complementary treatments (33.32%). Traditional/alternative and complementary treatments included: visiting centers for religious healing through a church or a priest, using holy water, Kitab (a written script tied on the arm or neck), slaughtering a sheep, fumigating (making excessive use of smoke by burning incense), Rukiya (Quranic-based religious treatment), beating, chaining and praying at home and/or at church. A preference for traditional/alternative and complementary treatment likely reflects misconceptions held in Africa about the perceived causes and prognosis of mental health conditions. These erroneous beliefs seem to be coupled with limited understanding of the biomedical and psychosocial causes of mental health conditions.

The second most frequently reported barrier to mental health services is stigma (25%). Tilahun et al. [25] found that most sampled caregivers experienced stigma. More specifically, 43.1% were concerned about being treated differently, 45.1% felt shame related to the mental health condition of their child, and 26.7% tried to keep the mental health condition of their child a secret. While professional and traditional/alternative treatments are not necessarily mutually exclusive, stigma was found to be *negatively* associated with seeking professional help [25]. Importantly, parents, especially those experiencing stigma, report resorting to traditional services for the mental health challenges of their children [22].

The third barrier to mental health services is a lack of knowledge or unfamiliarity with the mental health condition as reported in 25% of the studies included in our sample. This barrier is likely related to the first (preference for traditional services over more professional ones). For instance, in Rajcumar and Paruk [39], “92.4% of parents believed that reducing sugar or food additives were effective to reduce symptoms; 78.5% believed that treatments focusing on punishment reduced the symptoms; 67.1% thought that prolonged use of stimulant medications leads to increased addiction (i.e., drug, alcohol) in adulthood” (pg. 1).

Other parent-reported barriers to mental health services include: a low education level (25%), financial difficulties (16.67%), race (Black racial identity; 8.34%), a history of suicide attempts (8.34%), expectation that care services will be unavailable (8.34%), poor detection and assessment of mental health difficulties (8.34%), fear related to medication (8.34%), patient refusal to seek help (8.34%), parental denial of mental health difficulties (8.34%), residing in a rural area (8.34%), being a housewife or a farmer compared to being a merchant or occupying another profession (8.34%), delayed parental concern over their children’s mental health difficulty (8.34%), Coptic Christian religious identity (8.34%), and older age (older than 19 years; 8.34%).

Relatedly, these aforementioned barriers could be interrelated. Compared to parents with more years of education, uneducated parents and parents with fewer years of education (having less than 9 years of schooling) were approximately five times more likely to prefer traditional treatment options [32]. Additionally, higher maternal education appeared to be associated with greater knowledge of child development [22]. As for careers, housewives and farmers were 1.5 times more likely to prefer traditional treatment compared to merchants and other professionals [32]. Lastly, it is possible that parents occupying a lower socioeconomic status may have increased difficulty in offering their children frequent and consistent professional mental health care. This difficulty may in part be due to their inability to afford professional treatment or forego opportunities to generate income [26].

### Facilitators to mental health services

Although we were not originally interested in identifying facilitators to mental health services, as evidenced in our search terms, some of these facilitators could be pertinent from a research, clinical, or policy perspective. More specifically, a better understanding of facilitators to mental health services in an understudied context could represent an opportunity to better reach and service an underserved population. Facilitators to professional mental health services include having a referral by either a professional, a family member or teacher, more years of maternal education, having a job, a higher socioeconomic status, being part of a large family, and older age (older than 19 years old). Experiencing or having exposure to mental health difficulties also appears to act as a facilitator. This exposure could include having a different family member with mental health difficulties, or having family or former patients of a mental health treatment center provide information about mental health treatment. Other reported facilitators include: participating in ASD awareness campaigns and holding a positive attitude toward seeking psychological help.

## Discussion

Even though childhood and adolescent mental health conditions have been reported globally [41–43], they are far from being equally distributed [44]. Whereas most previous studies focused on high-income countries [20, 45], the few studies conducted in Africa focused on a few countries (most commonly South Africa) and favored an adult population. There is a paucity of quantitative comprehensive reviews pertaining to children and adolescents across Africa. Given the important treatment gap (between individuals in need of mental health services versus those who actually receive them), we were interested in identifying possible barriers to mental health services. Given the gatekeeper role parents usually play in providing their children access to mental health care, we were interested in gathering their perspectives.

In total, this scoping review pertained to six African countries: Ethiopia, Mali, Egypt, South Africa, Nigeria, and Tunisia. Treatments/services sought for these mental health challenges were diverse and spanned both professional services (e.g., psychiatric, psychologist) as well as traditional/alternative and complementary services (e.g., prayers and Kitab). Interestingly, the most frequented treatment centers were psychiatric hospitals. The reason why psychiatric hospitals were the most frequented treatment centers is likely attributed to the severity of the mental health condition and reserving mental health care for the most serious cases. This is an important finding that makes us question how many children are not receiving any help if their mental health conditions are not considered serious enough. The severity of mental health conditions should not be considered indicators of patients’ quality of life. Everyone suffering from mental health conditions deserves help and relief.

Perhaps most pertinent to this review is the finding that the most frequently reported barrier to seeking mental health care is a preference for traditional/alternative services. According to Abera, Robbins and Tesfaye [32], caregivers who believed that mental health conditions have underlying supernatural causes were 4.3 times more likely to prefer traditional treatments. The culturally relevant belief that psychological conditions can be explained by supernatural phenomena will likely inform the types of treatment or services sought. While some of these services are not necessarily harmful, other traditional/alternative treatments to mental health difficulties can be (e.g., chaining, beating, and restraining the child). In a study conducted by Gillespie-Lynch et al. [46] in Kenya and Nairobi, some participants described community members selling their farms to send their children to India for stem cell treatment. Stem cell treatment is not evidence-based [47]. The fact that parents go to such extents to address their children’s mental health challenges highlights the urgency of tackling mental health misconceptions in Africa. Relatedly, stigma acting as a barrier to mental health services is not surprising as evidenced by findings of studies in both high-income [20, 46] and low-income countries [48, 49]. However, what is pertinent is the fact that stigma seems to be associated with less understanding of the biomedical and psychosocial causes of mental health conditions and a greater propensity to seek traditional or alternative services [25]. Lastly and probably related to the first two barriers, lack of knowledge and awareness of mental health conditions also seems to act as a barrier to mental health services. This barrier relates to poor mental health literacy, which is not unusual in low-income countries [25] and in some high-income countries [50]. In some high-income countries, such as France, the inaccurate belief that poor parenting causes ASD, led to children being sequestered in hospitals [51]. Individuals with ASD in France have historically been far less likely to attend school and university, than students with ASD in countries with better autism services. This is likely due to difficulties accessing diagnoses and appropriate care [52]. Compared to some high-income countries with poor mental literacy that have and continue to make efforts to improve mental health knowledge [50], the results of this study suggest that some countries in Africa are lagging. These persistent erroneous mental health beliefs could be attributed to psycho-socio-cultural factors. This reduced access is probably exacerbated by economic constraints, limited educational opportunities, and communities that are predominantly lower in socioeconomic status [39, 53]. These findings shed light on the importance of public health campaigns, mental health training, and psychoeducation about mental health difficulties at the community and population level. The fact that the three most frequently reported barriers to mental health services and care are related speaks to the complexity of the issue. Based on the findings obtained in this review, we believe that future researchers, clinicians, and policy-makers should consider a two-level action plan.

The first level of the action plan pertains to the implementation of a local surveillance system for mental health conditions in multiple countries across Africa. This surveillance system is important given the dearth of studies reporting on mental health prevalence rates in Africa [1]. Much of the knowledge pertaining to childhood and adolescent mental health is generated in high-income countries [42, 43]. As was mentioned in the WHO (2013) report, there is a need to increase evidence grounded in low-resource settings. A better understanding of the prevalence rates of mental health conditions will help us better grasp the treatment needs and subsequent treatment gaps.

The second level of the proposed action plan is interventional. This intervention plan should target, amongst others, the reported barriers to mental health services identified in this review. To facilitate access to professional mental health services, improving peoples’ understanding of the biomedical and psychosocial causes of mental health conditions is key. Public awareness campaigns constitute a cost-effective and potentially far-reaching way of bridging the mental health knowledge gap. Training constitutes another way of filling this gap [54, 55]. Mental health training can perhaps address global disparities in access to mental health services by empowering local people in low-resource countries [12, 44, 56]. This training should favor a bottom-up approach to build upon existing knowledge held by parents and professionals in Africa. An example of such intervention includes an autism training offered by Gillespie-Lynch et al. [46] in Nairobi and Kenya. Participation in this training was associated with improved autism knowledge. Through offering such training, the hope is not only to increase mental health knowledge but also to decrease mental health stigma [57]. This finding is in line with [58] study. In their study, the researchers offered an autism training to educators in Canada and the US. Participation in the training was associated with increased autism knowledge and decreased stigma. In providing such mental health training, one needs to be respectful of the beliefs and preferences of the people from that community while advising them on safe and evidence-based interventions. Relatedly, given that traditional healers are often the first point or the only point of contact for some parents, providing training to these healers could help facilitate adequate and timely psychological referrals [59].

Interventions mediated by parents and other non-specialist providers also have the potential to significantly increase access to care [1, 57]. Given the complexity of some mental health conditions and to bypass the lack of available professionals in Africa, parental training could be offered online [57]. This training format could represent a cost-effective way of reaching people in rural areas, especially if they are provided in community libraries and centers. Lastly, the suggested recommendations will probably not be fully implemented without strong governmental leadership and support, adequate resources, and the commitment of key stakeholders (non-governmental organizations, governmental agencies). Even though some positive movement has been witnessed in some countries in Africa (e.g., The South African Mental Health Care Act), efforts remain unequally distributed and inconsistent.

## Limitations

This study has a number of limitations. Limiting the scope of the search to articles published over the past 10 years could have missed some articles. Some articles could not be retrieved online and gray literature was omitted. Studies favoring a qualitative approach were also excluded from this review. The results of such studies were therefore not reported. The large heterogeneity in sample sizes, methodology adopted, measurement tools, participant ages, place of residency, treatments, and services received, and where these services were received were all described differently across studies. The discrepant presentation of these variables across studies presented a challenge for the researchers to synthesize and describe. Additionally, understanding whether the researchers evaluated mental health symptoms or a mental health diagnosis was not always clear. Most of the studies included in this review originated in South Africa. While this over-representation of studies conducted in some African countries limits the generalizability of our findings, it also reflects the need to conduct studies in other African countries. Lastly, studies included in this review had a bias risk ranging between 17 and 22. These bias scores reflect the quality of the studies included in our review. While we were not comfortable with including studies that did not meet our inclusion criteria, we believe that future studies could broaden their scope to include lower-quality studies. More specifically, lower quality studies can have as much impact in establishing the context of the research topic as higher quality studies.

## Conclusion

The results of our study revealed that the three most frequently encountered barriers to accessing mental health services in Africa are: a preference for traditional/alternative and complementary treatments (33.33%), followed by stigma (25%), and a lack of knowledge/unfamiliarity with the mental health condition (25%). In terms of mental health services sought, the most frequently reported services were psychiatric treatments (25%), screening and diagnostic assessment (16.67%), as well as psychiatric and psychological consultations (16.67%). Africa’s 98.8% mental health treatment gap should serve as an alarm bell. We hope that this review will help shed light on mental health barriers and provide future generations with valuable information to help reduce the mental health treatment gap.

## Data Availability

The datasets generated and/or analyzed during the current study are available in the OSF repository, https://osf.io/eks6c/.

## Abbreviations

ADHD: Attention Deficit Hyperactivity Disorder
ASD: Autism Spectrum Disorder
BD: Bipolar Disorder
CD: Conduct Disorder
DD: Developmental Delays
HIV: Human Immunodeficiency Virus
ID: Intellectual Disability
MDD: Major Depressive Disorder
NR: Not reported
SUD: Substance Use Disorders
WHO: World Health Organization
Y: Years

## References

[1] World Health Organization (2013). Autism spectrum disorders & other developmental disorders from raising awareness to building capacity.

[2] National Academies of Sciences Engineering and Medicine. Adolescent development. Washington DC; 2019. p. 37–76. Available from: https://www.ncbi.nlm.nih.gov/books/NBK545476/.

[3] Babatunde GB, Van Rensburg AJ, Bhana A, Petersen I (2021). Barriers and facilitators to child and adolescent mental health services in low-and-middle-income countries: a scoping review. Glob Soc Welf.

[4] WHO MHGAPm. Scaling up care for mental, neurological and substance use disorders. 2008. 26290926

[5] Mclaughlin KA, Green JG, Gruber MJ, Sampson NA, Zaslavsky AM, Kessler RC (2012). Childhood adversities and first onset of psychiatric disorders in a National Sample of US Adolescents. Arch Gen Psychiatry.

[6] Patel V, Flisher AJ, Hetrick S, McGorry P (2007). Mental health of young people: a global public-health challenge. Lancet.

[7] Polanczyk GV, Salum GA, Sugaya LS, Caye A, Rohde LA (2015). Annual research review: a meta-analysis of the worldwide prevalence of mental disorders in children and adolescents. J Child Psychol Psychiatry.

[8] Fineberg NA, Haddad PM, Carpenter L, Gannon B, Sharpe R, Young AH (2013). The size, burden and cost of disorders of the brain in the UK. J Psychopharmacol.

[9] Whitney DG, Peterson MD (2019). US national and state-level prevalence of mental health disorders and disparities of mental health care use in children. JAMA Pediatr.

[10] Patel V, Kieling C, Maulik PK, Divan G (2013). Improving access to care for children with mental disorders: a global perspective. Arch Dis Child.

[11] Paula CS, Bordin IA, Mari JJ, Velasque L, Rohde LA, Coutinho ES (2014). The mental health care gap among children and adolescents: data from an epidemiological survey from four Brazilian regions. PLoS One.

[12] Tilahun D, Hanlon C, Araya M, Davey B, Hoekstra RA, Fekadu A (2017). Training needs and perspectives of community health workers in relation to integrating child mental health care into primary health care in a rural setting in sub-Saharan Africa: a mixed methods study. Int J Ment Heal Syst.

[13] Kieling C, Baker-Henningham H, Belfer M, Conti G, Ertem I, Omigbodun O (2011). Child and adolescent mental health worldwide: evidence for action. Lancet.

[14] Sankoh O, Sevalie S, Weston M (2018). Mental health in Africa. Lancet Glob Health.

[15] World Health Organization. Mental health atlas 2014–2015. 2015.

[16] Yoder HNC, Tol WA, Reis R, De Jong JTVM (2016). Child mental health in Sierra Leone: a survey and exploratory qualitative study. Int J Ment Heal Syst.

[17] Boulter E, Rickwood D (2014). Parents’ experience of seeking help for children with mental health problems. Adv Ment Health.

[18] Bussing R, Gary FA, Mills TL, Garvan CW (2003). Parental explanatory models of ADHD: gender and cultural variations. Soc Psychiatry Psychiatr Epidemiol.

[19] World Health Organization (2018). Global health estimates 2016: burden of disease by cause, age, sex, by country and by region, 2000–2016.

[20] Radovic A, Farris C, Reynolds K, Reis EC, Miller E, Stein BD (2014). Primary care providers’ beliefs about teen and parent barriers to depression care. J Dev Behav Pediatr.

[21] Turner EA, Jensen-Doss A, Heffer RW (2015). Ethnicity as a moderator of how parents’ attitudes and perceived stigma influence intentions to seek child mental health services. Cultur Divers Ethnic Minor Psychol.

[22] Erasmus S, Kritzinger A, Van der Linde J (2021). Onset of intervention for learners in autism-specific government-funded schools in South Africa. Int J Disabil Dev Educ.

[23] Pillay AL, Siyothula ET (2011). Intellectual disability examinations and social context variables among patients of low socioeconomic status. Percept Mot Skills.

[24] Schierenbeck I, Johansson P, Andersson L, Van Rooyen D (2013). Barriers to accessing and receiving mental health care in Eastern Cape, South Africa. Health Hum Rights.

[25] Tilahun D, Hanlon C, Fekadu A, Tekola B, Baheretibeb Y, Hoekstra RA (2016). Stigma, explanatory models and unmet needs of caregivers of children with developmental disorders in a low-income African country: a cross-sectional facility-based survey. BMC Health Serv Res.

[26] Ambikile JS, Outwater A (2012). Challenges of caring for children with mental disorders: experiences and views of caregivers attending the outpatient clinic at Muhimbili National Hospital, Dar es Salaam-Tanzania. Child Adolesc Psychiatry Ment Health.

[27] Divan G, Vajaratkar V, Desai MU, Strik-Lievers L, Patel V (2012). Challenges, coping strategies, and unmet needs of families with a child with autism spectrum disorder in Goa, India. Autism Res.

[28] Arksey H, O’Malley L (2005). Scoping studies: towards a methodological framework. Int J Soc Res Methodol.

[29] American Psychiatric Association. Diagnostic and statistical manual of mental disorders (5th ed). 2013.

[30] Moher D, Liberati A, Tetzlaff J, Altman DG, PRISMA Group* (2009). Preferred reporting items for systematic reviews and meta-analyses: the PRISMA statement. Ann Intern Med.

[31] Kmet LM, Cook LS, Lee RC. Standard quality assessment criteria for evaluating primary research papers from a variety of fields. 2004.

[32] Abera M, Robbins JM, Tesfaye M (2015). Parents’ perception of child and adolescent mental health problems and their choice of treatment option in southwest Ethiopia. Child Adolesc Psychiatry Ment Health.

[33] Abiodun O, Tunde-Ayinmode M, Ayinmode BA, Adegunloye OA (2011). Detecting child psychiatric disorders during routine clinic work: A pre-interventional study of primary care physicians in Ilorin, Nigeria. S Afr J Psychiatry.

[34] Bourgou S, Halayem S, Bouden A, Halayem MB (2012). Tunisian mothers' beliefs about their child's first psychotic episode. Encephale..

[35] Burnhams NH, Dada S, Myers B. Social service offices as a point of entry into substance abuse treatment for poor South Africans. Subst Abuse Treat Prev Policy. 2012;7:22. 10.1186/1747-597x-7-22. 10.1186/1747-597X-7-22 PMC3414793 22642796

[36] Girma E, Tesfaye M. Patterns of treatment seeking behavior for mental illnesses in Southwest Ethiopia: a hospital based study. BMC Psychiatry. 2011;11(1):138. 10.1186/1471-244X-11-138. 10.1186/1471-244X-11-138 PMC3170592 21859455

[37] Gobrial E (2012). Mind the gap: the human rights of children with intellectual disabilities in Egypt. J Intellect Disabil Res..

[38] Hussein H, Shaker N, El-Sheikh M, Ramy HA (2012). Pathways to child mental health services among patients in an urban clinical setting in Egypt. Psychiatr Serv.

[39] Rajcumar NR, Paruk S (2020). Knowledge and misconceptions of parents of children with attention-deficit hyperactivity disorder at a hospital in South Africa. S Afr Fam Pract.

[40] Sangare M, Fousso F, Touré A, Ghislan V, Traoré K, Coulibaly SP (2019). Health facility-based prevalence and potential risk factors of autism spectrum disorders in Mali. Afr J Neurol Sci..

[41] Chen YL, Chen WJ, Lin KC, Shen LJ, Gau SSF. Prevalence of DSM-5 mental disorders in a nationally representative sample of children in Taiwan: methodology and main findings. Epidemiol Psychiatr Sci. 2020;29:e15. 10.1017/S2045796018000793 PMC8061245 30696515

[42] Georgiades K, Duncan L, Wang L, Comeau J, Boyle M (2019). Six-month prevalence of mental disorders and service contacts among children and youth in Ontario: evidence from the 2014 Ontario child health study. Can J Psychiatry.

[43] Xu G, Strathearn L, Liu B, Bao W (2018). Prevalence of autism spectrum disorder among US children and adolescents, 2014–2016. JAMA.

[44] Gillespie-Lynch K, Daou N, Sanchez-Ruiz MJ, Kapp SK, Obeid R, Brooks PJ (2019). Factors underlying cross-cultural differences in stigma toward autism among college students in Lebanon and the United States. Autism.

[45] Hansen AS, Telléus GK, Mohr-Jensen C, Lauritsen MB (2021). Parent-perceived barriers to accessing services for their child’s mental health problems. Child Adolesc Psychiatry Ment Health.

[46] Gillespie-Lynch K, Mathaga J, Daou N, Muiruri J, Nerea Okello M, Green A, et al. Evaluating a training to improve autism knowledge and stigma in Kenya. International Meeting for Autism Research (INSAR); 2019.

[47] Riccio A. Autism in Kenya: a social, educational, and political perspective. Independent Study Project (ISP) Collection; 2011. https://digitalcollections.sit.edu/isp_collection/1203.

[48] Doumit MAA, Farhood LF, Hamady C (2018). Focus groups investigating mental health attitudes and beliefs of parents and teachers in South Lebanon: are they culturally determined?. J Transcult Nurs.

[49] Gearing RE, MacKenzie MJ, Ibrahim RW, Brewer KB, Batayneh JS, Schwalbe CS (2015). Stigma and mental health treatment of adolescents with depression in Jordan. Community Ment Health J.

[50] Saade S, Bockstal-Fieulaine B, Gillespie-Lynch K, Besche-Richard C, Boujut E, Johnson Harrison A, Cappe E. Evaluation of an Autism Training in a Much-Needed Context: The Case of France. Autism in Adulthood. 2023. 10.1089/aut.2022.0080. 10.1089/aut.2022.0080 PMC10468555 37663443

[51] Vautrin J. Rapport sur le vécu des autistes et de leurs familles en France à l’aube du XXIème siècle. Limoges, éditions Autisme France. 1994.

[52] Schofield H. France’s autism treatment ‘shame’. 2022. https://www.bbc.com/news/magazine-17583123.

[53] Dosreis S, Zito JM, Safer DJ, Soeken KL, Mitchell JW, Ellwood LC (2003). Parental perceptions and satisfaction with stimulant medication for attention-deficit hyperactivity disorder. J Dev Behav Pediatr.

[54] Gillespie-Lynch K, Bisson JB, Saade S, Obeid R, Kofner B, Harrison AJ, et al. If you want to develop an effective autism training, ask autistic students to help you. Autism. 2021;26:13623613211041006. 10.1177/13623613211041006 34472359

[55] Gillespie-Lynch K, Brooks PJ, Someki F, Obeid R, Shane-Simpson C, Kapp SK (2015). Changing college students’ conceptions of autism: an online training to increase knowledge and decrease stigma. J Autism Dev Disord.

[56] Harrison AJ, Long KA, Manji KP, Blane KK (2016). Development of a brief intervention to improve knowledge of autism and behavioral strategies among parents in Tanzania. Intellect Dev Disabil.

[57] Obeid R, Saade S (2022). An urgent call for action: Lebanon’s children are falling through the cracks after economic collapse and a destructive blast. Glob Ment Health.

[58] Saade S, Bean YF, Gillespie-Lynch K, Poirier N, Harrison AJ. Can participation in an online ASD training enhance attitudes toward inclusion, teaching self-efficacy and ASD knowledge among preservice educators in diverse cultural contexts? Int J Incl Educ. 2021:1–16.

[59] Sorsdahl K, Stein DJ, Grimsrud A, Seedat S, Flisher AJ, Williams DR (2009). Traditional healers in the treatment of common mental disorders in South Africa. J Nerv Ment Disord.

