# Correction: What barriers could impede access to mental health services for children and adolescents in Africa? A scoping review

**DOI:** 10.1186/s12913-024-11046-4

**Published:** 2024-05-01

**Authors:** Sabine Saade, Annick Parent-Lamarche, Tatiana Khalaf, Sara Makke, Alexander Legg

**Affiliations:** 1 Department of Psychology, American University of Beirut, P.O.Box 11-0236, Riad El-Solh/Beirut, 1107 2020 Lebanon; 2 Département de gestion des ressources humaines, Université du Québec à Trois-Rivières, 3351, boulevard des Forges, Trois-Rivières, QC G8Z 4M3 Canada; 3 Department of Psychology, CUNY John Jay College of Criminal Justice, 524 W 59th Street, New York, NY 10019 USA

**Correction to:** ***BMC Health Services Research*** **(2023) 23:348**

10.1186/s12913-023-09294-x

In this article, the author name Annick Parent-Lamarche was incorrectly written as Annick Parent Lamarche (where ‘Parent’ was incorrectly tagged as part of the given name instead of the family name). The author group has been updated above and the original article (including its metadata) has been corrected.

